# SV-STAT accurately detects structural variation via alignment to reference-based assemblies

**DOI:** 10.1186/s13029-016-0051-0

**Published:** 2016-06-18

**Authors:** Caleb F. Davis, Deborah I. Ritter, David A. Wheeler, Hongmei Wang, Yan Ding, Shannon P. Dugan, Matthew N. Bainbridge, Donna M. Muzny, Pulivarthi H. Rao, Tsz-Kwong Man, Sharon E. Plon, Richard A. Gibbs, Ching C. Lau

**Affiliations:** Structural and Computational Biology and Molecular Biophysics (SCBMB) Program, Baylor College of Medicine, Houston, TX 77030 USA; Texas Children’s Cancer Center, Baylor College of Medicine, Houston, TX 77030 USA; W. M. Keck Center for Interdisciplinary Bioscience Training, Houston, TX 77005 USA; Human Genome Sequencing Center, Baylor College of Medicine, One Baylor Plaza, N1621, Houston, TX 77030 USA

**Keywords:** Algorithm, Genome, Sequencing, Structural variation, Genotype, Translocation, Cancer

## Abstract

**Background:**

Genomic deletions, inversions, and other rearrangements known collectively as structural variations (SVs) are implicated in many human disorders. Technologies for sequencing DNA provide a potentially rich source of information in which to detect breakpoints of structural variations at base-pair resolution. However, accurate prediction of SVs remains challenging, and existing informatics tools predict rearrangements with significant rates of false positives or negatives.

**Results:**

To address this challenge, we developed ‘Structural Variation detection by STAck and Tail’ (SV-STAT) which implements a novel scoring metric. The software uses this statistic to quantify evidence for structural variation in genomic regions suspected of harboring rearrangements. To demonstrate SV-STAT, we used targeted and genome-wide approaches. First, we applied a custom capture array followed by Roche/454 and SV-STAT to three pediatric B-lineage acute lymphoblastic leukemias, identifying five structural variations joining known and novel breakpoint regions. Next, we detected SVs genome-wide in paired-end Illumina data collected from additional tumor samples. SV-STAT showed predictive accuracy as high as or higher than leading alternatives. The software is freely available under the terms of the GNU General Public License version 3 at https://gitorious.org/svstat/svstat.

**Conclusions:**

SV-STAT works across multiple sequencing chemistries, paired and single-end technologies, targeted or whole-genome strategies, and it complements existing SV-detection software. The method is a significant advance towards accurate detection and genotyping of genomic rearrangements from DNA sequencing data.

**Electronic supplementary material:**

The online version of this article (doi:10.1186/s13029-016-0051-0) contains supplementary material, which is available to authorized users.

## Background

Structural variants (SVs) such as deletions, duplications, inversions, and translocations are implicated in a wide range of human diseases and are used as diagnostic and prognostic markers in lymphomas, leukemias, and sarcomas. For example, detection of interchromosomal translocations routinely guides the choice of treatment in pediatric patients with B-lineage acute lymphoblastic leukemia (B-ALL). Traditionally, these translocations [t(4;11), t(12;21), t(1;19), and t(9;22)] are detected with low-resolution methods such as fluorescence *in situ* hybridization, leaving them uncharacterized at the base-pair level, and limiting our understanding of their biological impact. Targeted deep-sequencing technologies provide a potentially rich source of information with which to detect breakpoints of clinically relevant genomic rearrangements [1–4], but accurate prediction of SVs with existing informatics tools remains challenging [5–7].

The most widely available deep-sequencing instruments provide the sequences of nucleotides (reads) along both (paired) ends of size-selected fragments of sheared DNA. Some leading algorithms (e.g. BreakDancer) use prior knowledge of this size distribution plus the mapped locations and orientations of the read pairs in the reference genome to detect SVs. This is called the “paired-end” method (PE). Other leading algorithms (e.g. CREST) discard paired information. Given an unpaired read, these algorithms typically search for non-overlapping sub-sequences mapping uniquely to different locations (breakpoint regions) in the reference genome [8–11]. This approach is commonly known as the “split-read” method (SR). Given the limited accuracies of PE and SR and the computational challenges of *de novo* assembly (DN), we explored methods for quantifying support for nucleotide-resolved breakpoints of SVs without PE, SR, or DN and applied our approach to recover SVs from target-enriched deep-sequencing data. Next, we appended our approach to PE for analysis of whole-genome sequencing data.

## Implementation

### Generate library of candidate SVs

**Fig. 1:**
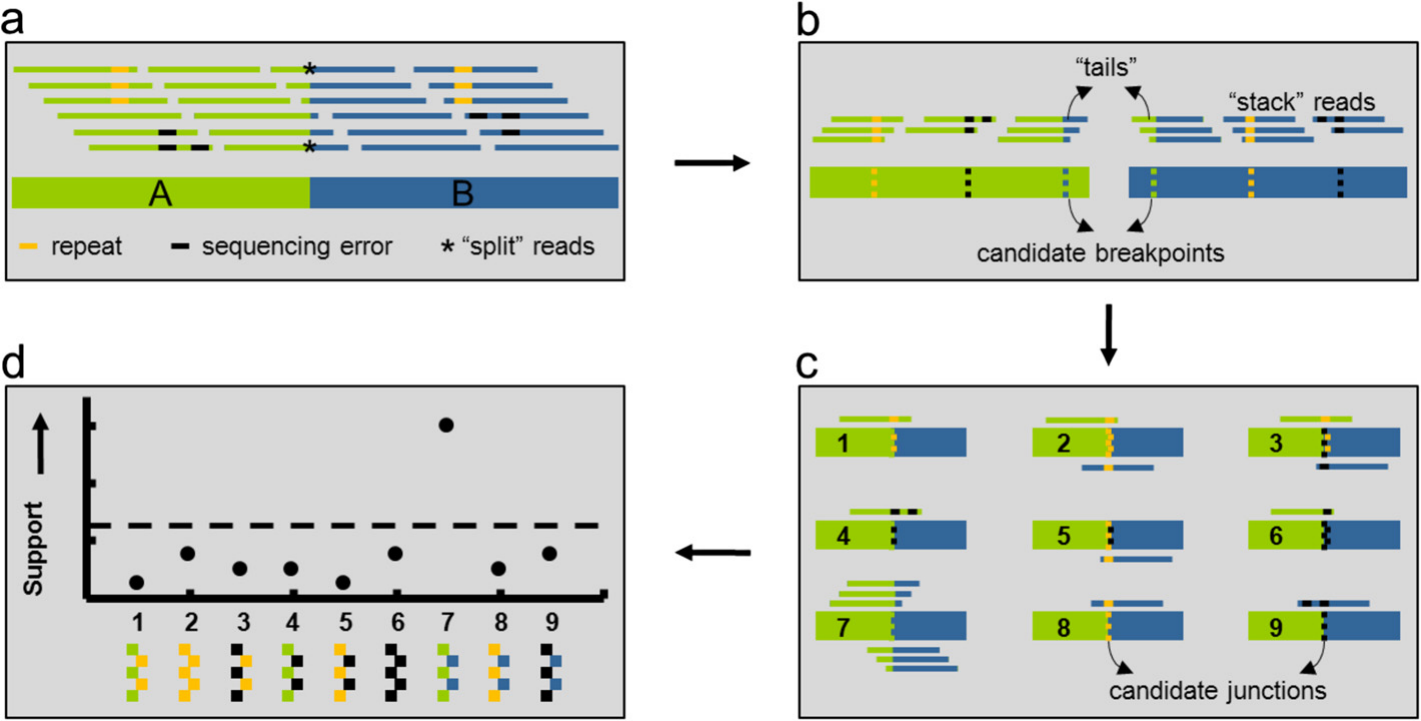
Use of chimeric and split reads to detect structural variation. Structural variation in the sample is depicted in box 1**a** as a fusion between genomic regions A (*green*) and B (*blue*). Sequence differences in the sample come from structural variation, repetitive sequence (*orange*), and base substitutions due to sequencing errors and SNPs (*black*). In box 1**b**, each group of partially aligned reads, or “stack,” corresponds to a candidate breakpoint located at shared end (left: *orange*, *black*, and *blue*; right: *green*, *yellow*, and *black*). Pairwise combinations of breakpoints form a library of candidate junctions (box 1**c**). All stacked reads are aligned to the library and are used to assess their support for the candidate junctions. A read aligned to a candidate provides support equal to the product of the length of the “tail” and total alignment quality. The total support for each candidate junction (box 1**d**) is the sum of supports from the stacked reads aligned to it

Figure 1 illustrates how in addition to split reads (*), other chimeric reads that map to only one genomic location can provide corroborative evidence for a SV connecting the breakpoint regions A (green) and B (blue) in the test sample (box 1a). In the first step of our approach, all reads are aligned to the reference genome. Reads with repetitive sequence (orange), DNA sequencing errors (black), or support for SVs (green & blue) align partially to the reference genome. Recurrent alignment stop or start coordinates indicate candidate breakpoints (box 1b). Bases aligned to the reference comprise the “stacks” while the remaining unaligned (soft-clipped) bases make up the “tails.” Next, candidate breakpoint regions are paired with each other to form a sequence “library” of candidate junctions (box 1c).

### Measure support for each candidate SV

Stack reads are then aligned to the library of candidate SVs, and evidence for each candidate junction (*C*) is calculated based on 1) the number of bases in the tails aligned to the partner region, and 2) the quality scores of the alignments. Specifically, the support (*S*) for each *C* is the summation of the product of these two values in all the stacked reads aligned to *C* according to the following equation:

$$ S={\displaystyle \sum_{i=1}^n}\kern0.28em {Q}_i \min \left\{{l}_{A,i},{l}_{B,i}\right\} $$

where *Q*_*i*_ is the quality score and min{*l*_*A,i*_,*l*_*B,i*_} is the lesser of the number of bases aligned to the i-th read (*i = 1,2,…,n*) in A or B.

Based on this strategy, we developed a software tool for Structural Variation detection by STAck and Tail (SV-STAT). The tool predicts the presence of structural variations in test samples relative to a reference genome. SV-STAT quantifies the amount of evidence for junctions of SVs at base-pair resolution and predicts SVs scoring above a user-defined threshold of support (box 1d).

## Results and Discussion

### Evaluation on targeted deep-sequencing data

We applied SV-STAT to Roche/454 sequencing data acquired from a custom hybridization array spanning 8 genomic regions recurrently rearranged in B-ALL (total of 1.3 Mb; see Additional file 1 for details). This array was applied to diagnostic samples of three patients with B-ALL (4, 65C, and 96C). Given only the sequence aligned to the reference genome, and the canonical orders and orientations of the breakpoint regions in the translocations (in Additional file 1: Figure S1), SV-STAT identified base-pair resolved breakpoints of reciprocal t(1;19) and t(4;11) translocations in samples 65C and 4, respectively. This result agreed with the cytogenetic analysis; however, no translocation was identified by SV-STAT in sample 96C, which was diagnosed with t(4;11)(q21;q23). We adjusted the input to SV-STAT to consider candidate inversions within the q arm of chromosome 11, which revealed a 1 Mb inversion connecting lysine-specific methyltransferase 2A (*MLL*) and ubiquitin specific peptidase 2 (*USP2*), a novel fusion partner for *MLL*. Overall, our analysis showed a total of five SVs, all of which were confirmed by amplification of genomic DNA across the junctions with polymerase chain reaction (PCR; in Additional file 1: Figure S9).

To compare SV-STAT against the leading algorithms for mapping assembly of SVs, we first tried to detect SVs in the same dataset with CREST, which uses local *de novo* assembly of reads from candidate breakpoint regions followed by split-read mapping to match partnering breakpoints [10]. CREST identified four of the five SVs identified by SV-STAT, and no additional SVs. Derivative chromosome 11 from the t(4;11) reciprocal translocation in sample 4 was not detected by CREST. R453Plus1Toolbox, a split-read mapping assembler trained to detect balanced translocations in target-enriched unpaired 454 deep-sequencing data [11], identified the same set of SVs as SV-STAT.

We used simulated target-enriched deep-sequencing data to evaluate further the predictive accuracies of each algorithm. These data were generated (see Supplemental methods in Additional file 1) based on translocations previously detected in B-ALL cases by conventional methods (e.g. Sanger sequencing). First, we divided the simulated data into a training set (4 samples; 7 translocations) and a test set (23 samples; 31 translocations). We used the training set to define the threshold, above which SV-STAT would predict SVs. This threshold (2.985045) was the average between the support scores of the lowest-scoring true positive (3.02119) and the false positive immediately below it (2.9489) (in Additional file 1: Table S3). We also trained SV-STAT to collapse similar predictions into a single SV (see “SV-STAT post-processing” in Additional file 1). Given the same training data, input parameters for CREST were also chosen for highest predictive accuracy. R453Plus1Toolbox did not accept any optional parameters.

Next, we used the algorithms to predict which of the four possible types of translocations (t(1;19), t(4;11), t(9;22), or t(12;21)) was present in 23 additional test samples. None of the algorithms produced a false positive. SV-STAT successfully predicted the translocation type correctly for all 23 samples, which was significantly more accurate than the predictions of both R453Plus1Toolbox (19/23 (83 %); *p* < 0.05), and CREST (18/23 (78 %); *p* < 0.005).

**Fig. 2:**
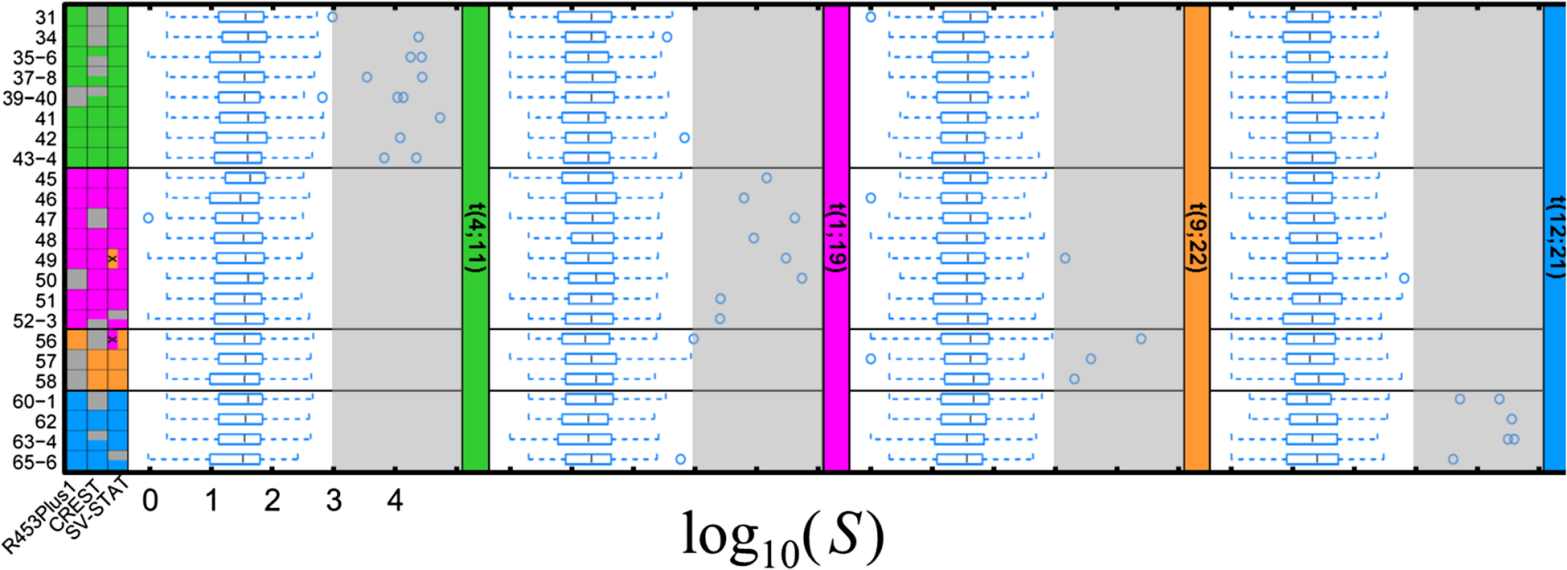
SV-STAT is more accurate than alternative methods for determining base-pair resolved breakpoints of translocations given unpaired Roche/454 sequencing data simulated from DNA fusions previously reported in pre-B ALL cases. Samples are arrayed in rows colored for translocations t(4;11) (*green*), t(1;19) (*purple*), t(9;22) (*orange*), and t(12;21) (*blue*). The first three columns are predictions of SVs from R453Plus1Toolbox [15], CREST [14], and SV-STAT. *Grey* indicates a false negative, or non-predicted translocation. A color with an “X” through it indicates a false positive, or wrongly-predicted translocation. Columns of boxplots indicate support (log10(S)) for candidate junctions, one column per type of SV. *Black* vertical dashes indicate median, rectangles indicate the interquartile (25–75 %) range, and upper and lower whiskers represent the boundaries of the 90 % and 10 % percentiles, respectively. Shaded regions indicate sufficient support for SV-STAT to predict SVs

At the level of the individual translocations, there were 31 translocated and 153 normal chromosomes (23 samples and 8 chromosomes per sample). SV-STAT achieved a sensitivity and positive predictive value (PPV) of 29/31 (93.5 %; Fig. 2). If the user chooses a threshold of 100 % PPV in SV-STAT, sensitivity would be 90.3 %. Translocations 52 and 65 in samples 52-3 and 65-6 were not predicted by SV-STAT because the corresponding junction-spanning reads aligned to candidate junctions with only one correct breakpoint region. However, both of these translocations occurred in samples with two reciprocal rearrangements, and in each case SV-STAT correctly predicted the second SV. Moreover, using BLAST instead of BWA to align stacked reads to libraries in samples 52-3 and 65-6 produced the correct alignments for translocations 52 and 65. Predictions of incorrect translocation types in samples 49 and 56 were accompanied with higher-scoring predictions of correct types of translocations (in Additional file 1: Table S4). In comparison, CREST’s sensitivity and specificity were 20/31 (64.5 %) and 20/20 (100 %), respectively, while R453Plus1Toolbox also achieved 100 % PPV with a sensitivity of 26/31 (83.9 %).

### Application to Illumina paired-end whole-genome sequencing data

While SV-STAT predicted SVs in Roche/454 data more accurately than CREST within the B-ALL breakpoint regions, the relative performances when applied to Illumina paired-end sequencing data genome-wide remained unknown. To perform the comparison between SV-STAT and CREST (R453Plus1Toolbox did not accept Illumina data), first we collected and aligned whole genome sequencing data [12] from a set of 6 childhood cancer patients enrolled in an ongoing familial cancer study [13]. In order to complete a whole-genome analysis by SV-STAT, we 1) limited the combinatorial space to pairs of candidate breakpoints identified by BreakDancer, 2) obtained the orders and orientations of the flanking regions as suggested by pairs of discordant reads within 1 Kb, 3) used a linear function to adjust SV-STAT’s score for variable coverage, and 4) excluded highly repetitive regions with excessive coverage. Otherwise, SV-STAT proceeded without significant modification.

SVs predicted by SV-STAT and CREST calls were compared using a window of 500 bp on either side of the SV event. Custom filters were applied to the raw outputs of CREST (see Additional file 1). The average number of calls per tumor sample was roughly similar (SV-STAT: 673+/-91, CREST: 750+/-170), as was the average intersection of SV events (SV-STAT: 85 %+/-2 %, CREST: 86 %+/-3 %). Of the SV events called solely by SV-STAT (*n* = 606), the deletions (*n* = 414) and insertions (*n* = 104) were spread evenly across all chromosomes (fraction of SVs/fraction of genome: 1.0+/-0.3). We used Integrated Genome Viewer [14] to randomly inspect events called uniquely by each algorithm, and found consistent patterns. Specifically, CREST did not consider paired-end information; therefore it tended to miss events with the bulk of the soft-clipping at only one of the breakpoints. Similarly, SV-STAT suffered from false negatives within repetitive regions where the majority of the reads aligned with single-nucleotide mismatches instead of soft-clipping.

While the accuracy demonstrated by SV-STAT is compelling, we have not addressed the detection of novel insertions longer than or approaching the lengths of the analyzed reads. Furthermore, the time complexity of SV-STAT’s underlying algorithm is polynomial, as opposed to the linear time complexities of split-read and paired-end methods. However, the algorithm is readily appended to paired-end analysis, and, in on-going studies, we routinely deploy it for concurrent analysis of larger numbers (>100) of whole-genomes. Furthermore, we built SV-STAT with usability and extensibility in mind. The code is freely available with unit tests, and the software performs checks for its minimal dependencies when launched without parameters.

With further development, we expect to generalize SV-STAT for routine genotyping and discovery of clinically relevant SVs across a wider range of human diseases. In particular, we will extend the software for capability to distinguish germline from somatic SVs. Complex SVs such as those generated by replication-fork stalling [15] where multiple breakpoints occur in close proximity could also be detected with recursive application of SV-STAT when additional soft-clipping remains following alignment of the stacked reads to the SV. Lastly, as a timesaving measure for this study, we used BreakDancer for the initial pairs of candidate breakpoints, but we will remove this dependency in the future, relying instead on the discordant read pairs obtained directly from the alignments.

## Conclusions

Similar approaches to measure support for candidate junctions in unpaired deep-sequencing data were reported [16–18]. SV-STAT extends these methods by adjusting a chimeric read’s support of an SV by 1) the number of its soft-clipped bases and 2) the quality of its alignment to the junction. Our results demonstrate the current version of SV-STAT is valuable in conjunction with DNA sequencing and existing tools for accurate genotyping and discovery of recurrent and novel SVs, respectively. In particular, SV-STAT’s scoring metric is applicable to alignments of test reads given any collection of suspected SVs, regardless of the method of assembly. Furthermore, detecting the inversion in 454/Roche data and the insertions and deletions genome-wide in Illumina data illustrates the capability of SV-STAT to interrogate a wide range of target sizes and to predict a diversity of candidate junction types across multiple platforms.

### Availability and requirements

- Project name: SV-STAT
- Project home page: https://gitorious.org/svstat/svstat
- Operating system(s): Unix-based
- Programming language: Perl, Python, and bash
- Other requirements: cdbfasta, bwa, picard, samtools, bioperl, and bedtools
- License: GPLv3

## Additional file

Additional file 1: Supplemental implementation, methods, figures, and tables. (DOCX 1382 kb)
