# Supplementary material for: SV-STAT accurately detects structural variation via alignment to reference-based assemblies
Source: Source Code Biol Med. 2016 Jun 18;11:8. doi: 10.1186/s13029-016-0051-0 (PMC4913042; doi:10.1186/s13029-016-0051-0)
Supplement: Additional file 1: — Supplemental implementation, methods, figures, and tables. (DOCX 1382 kb) [file 13029_2016_51_MOESM1_ESM.docx]

# Supplemental implementation

## SV-STAT applied to detection of recurrent SVs in pediatric B-ALL

We considered the set of alignments between a query ($q$) and a subject ($s$), where the query was the sequence of base pairs (read) of a fragment of DNA, and the subject was the reference genome. Multiple alignments were allowed per query as determined by the alignment program. The read length ($q_{l}$) referred to the number of base pairs in the query, while the start and end positions of an alignment within the read (local coordinates) were $q_{s}$ and $q_{e}$, respectively. In the reference, the first and last base pairs of an alignment were referred to as $s_{s}$ and $s_{e}$, respectively. Reads aligning to the opposite (“-”) strand of the reference were reverse complemented before local coordinates were reported. Alignments with $q_{s}=1$ and $q_{e}=q_{l}$ were ignored because the read aligned to the reference across its full length. If $q_{s}>1$ then $s_{s}$ was labeled with “start”. Similarly, if $q_{e}<q_{l}$ then $s_{e}$ was labeled with “end.” Multiple labels of the same type (start or end) at a coordinate in the reference indicated a candidate breakpoint. Candidate breakpoints of types start and end corresponded to “reverse” and “forward stacks” of reads, respectively as illustrated in Figure S7c. By default, we kept only those candidate breakpoints within the canonical B-ALL breakpoint clusters, and separated candidate breakpoints into four groups corresponding to breakpoint regions for t(4;11), t(12;21), t(1;19), and t(9;22).

Separately for each translocation type, DNA sequences were retrieved from the reference for the 500 base pairs (bp) preceding or following breakpoint coordinates of forward and reverse stacks, respectively. The ordering and orientation with which breakpoint regions were concatenated to form a candidate junction depended on the chromosomal arms involved in the translocation event. Let us consider the event provided in Figure S7 between the q arms of two chromosomes, chrA and chrB. Following t(A;B)(q;q), derivative chromosome A (derA) is modeled by a forward stack from chrA followed by a reverse stack from chrB. Similarly, derB is modeled by a forward stack from chrB followed by a reverse stack from chrA. These candidate junctions generated by SV-STAT are shown next to their corresponding derivative chromosomes in Figure S7d, which also indicates how SV-STAT generalizes to other types of interchromosomal SVs. For example, we used t(A;B)(p;q) derA to model der12 from t(12;21)(p13.2;q22.1) (left-hand side of lower-right quadrant in Figure S7d). Candidate junctions for der12 were generated by concatenating the reverse complement of sequence from a reverse stack from the q arm of chr21 (chrB; blue) to sequence from a reverse stack on the p arm of chr12 (chrA; green), in that order. Candidate junctions for the reciprocal SV, der21 (left-hand side of upper-left quadrant in Figure S7d), joined a forward stack from chr21 to the reverse complement of a forward stack from chr12, in that order.

Candidate junctions for inversions and all other types of intrachromosomal SVs [24] are modelled using combinations of forward and reverse stacks as shown in Figure S8 (upper-left and lower-right quadrants). SV-STAT accepts as input a list of candidate SVs. Each candidate SV is defined by the genomic coordinates and orientations (chr:coord:ori) of its pair of candidate breakpoint regions (e.g. chrA:coordA:oriA, chrB:coordB:oriB). For targeted analysis, we recommend a buffer size large enough to accommodate any uncertainties in the known breakpoint regions (up to 250 Kb in B-ALL). For detection of SVs genome-wide, where paired-end analysis by BreakDancer provides the list of candidates, we typically used a buffer size of 1000 base pairs.

A quality control filter considered reads from the paired stacks to remove candidate junctions unlikely to garner significant support. For example, a stacked read with few unaligned base pairs beyond its breakpoint (a short “tail”) is unlikely to contribute a significant amount of support to any candidate junction. Based on this principle, candidate junctions were rejected if 1) neither stack contained a read with a tail longer than four bp, or 2) the sum of tail lengths from reads in the paired stacks was less than 9. Candidates were indexed with BWA using the Burrows-Wheeler transform - Smith Waterman algorithm (-a bwtsw) with a maximum of three million candidates per index. If this step failed due to too few candidates, the “IS” (-a is) indexing option was used instead. Stacked reads were then aligned to the library of candidates with BWA-SW.

## Scoring metric for SV-STAT

Support for a candidate junction ($C$) was summed over the $n$ stacked reads ($R_{1},R_{2},...,R_{n}$) aligned to it. The $i$-th read ($i=1,2,...,n$) aligned to $C$ with quality score $Q_{i}$. The boundary in the candidate between breakpoint regions $A$ and $B$ was fixed in the library-creation step; therefore alignment coordinates along $C$ provided the number of bases in regions $A$ ($l_{A,i}$) and $B$ ($l_{B,i}$) spanned by the $i$-th read. Total support ($S$) for $C$ was defined as the product of the length of the “tail” and alignment quality summed over the junction-supporting reads. SV-STAT asserted the presence of the junction in the test sample if total support for the candidate exceeded the threshold identified during training ($S$ > 2.985045; see Tables S3 and S4).

## SV-STAT post-processing: Cluster candidate junctions by distance and support

Given breakpoints at physical locations $i$ and $j$ in regions $A$ and $B$, respectively, candidate junctions $C_{1}=$($A_{i,1},B_{j,1}$) and $C_{2}=$($A_{i,2},B_{j,2}$) with support scores $S_{1}$ and $S_{2}$ were merged if they were close to each other and well-supported. All pairwise cumulative supports ($S_{1}+S_{2}$) were determined, and pairwise distances were defined in Euclidian space ($\sqrt{{(A_{i,1}-A_{i,2})}^{2}+{(B_{j,1}-B_{j,2})}^{2}}$). Candidate junctions $C_{1}$ and $C_{2}$ were collapsed into the candidate with greater support if pairwise distance and the z-score of cumulative support met conditions identified during training (pairwise distance ≤ 20; z-score ≥ 2.27).

# Supplemental Methods

## Patient samples

We collected bone marrow samples from 3 de-identified patients with pediatric B-lineage acute lymphoblastic leukemia (B-ALL) using materials discarded by the clinical cytogenetics laboratory at Texas Children’s Hospital. Samples were chosen based on their known cytogenetics profile. The following are the cytogenetic diagnosis of each case: Sample 65C (46,XX, t(1;19)(q23;p13)); Sample 96C (46,XY, t(4;11)(q21;q23),+8); Sample 4 (46,XX, t(4;11)(q21;q23) [19]/46,XX[1].nuc ish(MLLx2)(5'MLL sep 3'MLLx1)[149/200]).

## DNA preparation

Whole bone marrow was cultured overnight in MarrowMax complete media (Invitrogen, Carlsbad, CA, USA), harvested and treated with 0.075M potassium chloride and fixed in carnoy’s fixative (3 parts methanol : 1 part glacial acetic acid). The resulting pellets were stored at -20°C. Upon DNA extraction, pellets were gently washed twice in ice-cold phosphate buffered saline and then incubated in 20 μL proteinase K overnight at 56°C on a rotary shaker. DNA was isolated from the cell extracts using QiaAmp columns according to manufacturer’s instructions (Qiagen, Hilden, Germany).

## DNA enrichment by hybridization and massively parallel sequencing

Arrays were ordered and designed by Roche-Nimblegen using ~385K probes to tile the target region. The target for enrichment was: March 2006 human genome assembly (hg18 [1]) chr1:162870000-163070000, chr11:117815000-117915000, chr12:11871000-11971000, chr19:1544000-1594000, chr21:35135000-35385000, chr22:21790000-21990000, chr4:88095000-88295000, and chr9:132560000-132760000. Twenty μg of genomic DNA was nebulized at 35 psi for 1 minute to an average size of 700 bp (range 500-900 bp). The nebulized DNA was purified using Zymo-Spin columns (Zymo Research, Irvine, CA, USA) and run on an Agilent Bioanalyzer 2100 DNAChip 7500 (Agilent Technologies, Santa Clara, CA, USA) or on a gel to determine the fragment size. The fragmented DNA was then polished and 5' phosphorylated using T4 DNA polymerase and T4 polynucleotide kinase. NimbleGen linkers (gsel3 and gsel4; Roche-Nimblegen, Madison, WI, USA) were ligated using T4 DNA ligase. Five μg of the pre-capture library was hybridized to the arrays at 42^o^C for 68 hours according to the NimbleGen array user’s guide. Five μg of amplified captured DNA was used to prepare DNA libraries for the Roche/454 platform [2] following standard protocols from the vendor [3, 4].

## Whole Genome Illumina Paired End Sequencing for Comparison of SV-STAT to CREST

CREST version 0.0.1 [28] was run on default parameters to generate raw output. The filtered call set was generated using empirically developed filters from consistent use and performance of CREST as follows: (1) require a BreakDancer supporting SV event within 600bp of a CREST SV event, (2) require at least 2 soft clips on either side of the SV event and (3) require at least 10 reads total coverage on either side of the breakpoint. BreakDancer (BreakDancerMax-1.1r112) [29] was run with all default parameters apart from a more stringent quality score (>=50). Window sizes from 10-1000bp were assessed to determine an optimal representative window of 500bp. Briefly, the SV comparison approach first separates within-chromosome events from translocation events, then numerically orders both call sets as follows: if the event is within chromosome it requires posA<posB and if the event is a translocation then chrA<chrB. The boundaries of the supporting callset are increased by the window size on either side of the event, and query calls must overlap by at least 1bp within both windows. Two comparative analysis were preformed: (1) Filtered SV-STAT to unfiltered CREST, and (2) filtered CREST to unfiltered SV-STAT.

## Process overview: simulate deep-sequencing data with coverage, read length, and base quality distributions modeled after unpaired Roche/454 sequencing of target-enriched DNA libraries

We built FASTA source files corresponding to DNA from individuals harboring translocations previously reported in patients with B-ALL. Reference sequences were added as needed to obtain a diploid target, or “sample.” Given the FASTA file for a sample, and a distribution of lengths, flowsim version 0.3 [14] simulated approximately 2.5x10^6^ fragments of DNA. A fragment was accepted or ignored according to its probability of “capture,” which we estimated using empirical coverage distributions. Flowsim then generated a flowgram, or “read” for each captured fragment of DNA.

## Generate FASTA source files of previously reported fusions in pre-B ALL

The four most-common types of prognostic translocations in pediatric B-lineage acute lymphoblastic leukemia (B-ALL) are t(12;21) TEL-AML, t(1;19) E2A-PBX, t(9;22) BCR-ABL, and t(4;11) MLL-AF4. Models of 38 previously reported [5-12] DNA fusions as curated in TICdb [13] were generated in FASTA format. Specifically, we used reference sequence from the boundary of the target in the first breakpoint region to the last base before the fusion. Subsequent bases spanned the partnering breakpoint region from the first base following the fusion to the end of the target. Ordering of the breakpoint regions and their orientations in the junctions depended on the translocation type as illustrated in Figure S1. By convention, a derivative chromosome was numbered according to its centromere’s chromosome of origin, and a junction’s sequence modeled the “+” strand of the derivative chromosome. When available in TICdb, reciprocal fusions (e.g. derivative chromosomes 4 and 11 for t(4;11)) were included together in a sample. Eight of 23 samples contained reciprocal fusions. Reference sequences were added as needed to a sample’s FASTA file in order to model a genome diploid for the regions in the target. Physical locations – coordinates mapped to the March 2006 reference human genome (hg18 [1]) – of breakpoints of the modeled translocations are reported in Table S2.

## Simulate region-specific enrichment of fragments of DNA

The clonesim module of flowsim was used to generate DNA fragments from the FASTA files. We used a weighted mixture of four fragment length distributions in order to better approximate the read length distribution observed experimentally (Figure S2). Eight percent of fragments were drawn from a lognormal length distribution ($\mu$ = 3.9; $\sigma$ = 0.2), 27% from a uniform distribution ($a=$ 65; $a=$ 400), 55% from a normal distribution ($\mu$ = 390; $\sigma$ = 55), and 10% from another lognormal distribution ($\mu$ = 5.7; $\sigma$ = 0.3). The reference coordinates spanned by a simulated fragment were incorporated into its FASTA header.

We filtered fragments with a custom software tool in order to simulate a coverage distribution similar to experimental results in samples 4 and 96C. Fragments were accepted or rejected as a function of the reference coordinates they spanned. Average coverage between samples 96C and 4 was computed for each base $(C_{coord})$, and the maximum $C_{coord}$ across the capture target was stored as $C_{max}$. Capture probability ${(p}_{capture})$ for a fragment of length $l$ with physical start and end coordinates $f_{s}$ and $f_{e}$, respectively was approximated by:

|  | $p_{capture}=\frac{\sum_{i=f_{s}}^{f_{e}} C_{i}}{C_{max}l}=\frac{AUC}{C_{max}l}$ |  |
| --- | --- | --- |

As illustrated on a plot of average coverage as a function of genomic coordinate (Figure S3), $p_{capture}$ is the area under the curve (AUC) within the span of the fragment $(l)$ divided by the area of the rectangle defined by $l$ and $C_{max}$.

We treated fragments spanning a junction differently in order to approximate incomplete “hybridization.” Given a fragment spanning breakpoint regions $A$ and $B$ (Figure S4), the capture probability was approximated by:

|  | $p_{capture}=\max\left\{ \frac{{AUC}_{A}}{C_{max}l_{A}},\frac{{AUC}_{B}}{C_{max}l_{B}} \right\}$ |  |
| --- | --- | --- |

the maximum of two hybridizations where $l_{A}$ and $l_{B}$ are the numbers of base pairs in the chimeric fragment corresponding to regions $A$ and $B$, respectively. Representative simulated and experimental coverage values are shown in Figure S5.

## Simulate pyrosequencing of captured DNA fragments: Base calls and quality scores

We used flowsim to estimate the base calls and qualities expected from pyrosequencing of the captured fragments of DNA [14]. Briefly, flowsim simulated emission of photons in proportion to the number of consecutive complementary nucleotides in the DNA as a function of the base-wise (“A”, “G”, “T”, or “C”) addition, or “flow,” of nucleotide substrate across primed DNA/polymerase assemblies. Such “flowgrams” for each fragment were generated with kitsim, mutator, and flowsim. The “generation” parameter for flowsim was set to “Titanium.” The base calls and initial qualities were generated using the flower module. Initial base qualities between 20 and 60 were rescaled to between 20 and 40 in order to more closely match the experimental distribution (Figure S6).

## Data pre-processing

Roche/454 DNA sequencing reads in FASTQ [15] format were pre-processed to remove reads with errors such as mismatches in lengths of strings for DNA sequence and base quality [16]. FastQC [17] was used to visualize read distributions such as lengths, base qualities, and k-mer content. NimbleGen adapter sequences were removed. Those reads passing quality control were aligned to the March 2006 human genome assembly (hg18 [1]) by Burrows-Wheeler Aligner Smith-Waterman (BWA-SW version 0.5.9-r16 [18]). We used samtools (version 0.1.18 r982:295 [19]), cdbfasta (version 0.99 [20]), bamToBed (version 2.11.2 [21]), Bio::DB::Sam (BioPerl package version 1.30 [22]), GNU coreutils version 8.4, GNU grep version 2.6.3, and GNU Awk version 3.1.7 to manipulate alignments for sorting, filtering, indexing, and retrieval. PCR-duplicate reads were removed with java version 1.6.0_20 and picard-tools version 1.40 [23]. R453Plus1Toolbox required removal of “chr” from all chromosome names in its annotations. Otherwise, the same alignment files were used as input to SV-STAT, CREST, and R453Plus1Toolbox.

## Parameters used in CREST and R453Plus1Toolbox

We used the CREST suite, version 0.0.1. We supplied the --nopaired parameter to CREST.pl and extractSClip.pl. Additional parameters provided to CREST.pl were -l 250, --min_sclip_reads 2, and --min_one_side_reads 2. We used R453Plus1Toolbox_version 1.4.0 under R version 2.14 with reference genome hg18 version 1.3.17 from BSgenome [25].

## Evaluate predictive accuracies of algorithms

Successful prediction of a translocation for all algorithms required detection of at least two reads supporting each breakpoint. SVs identified outside the B-ALL breakpoint regions, and SVs connecting non-canonical pairs of breakpoint regions were ignored. Successful classification of the SV [t(4;11), t(1;19), t(9;22), or t(12;21)] present in the patient’s sample required detection of at least one correct translocation. If translocations of more than one type of SV were predicted in a sample then only the highest-scoring type was considered. Differences between predictive accuracies were evaluated for significance by determining the probability with which the greater number of successes or more would occur by chance alone (assuming a binomial distribution) given the lesser success rate.

# Supplemental Figures

## Figure S1 - Ordering and orientation of breakpoint regions by type of translocation

Chromosomes are illustrated with mapping coordinates increasing from top to bottom on the forward (“+”) strand. The chromosomes’ p (from the French *petit*, small) and q (q follows p in the Latin alphabet) arms are above and below the centromeres (orange), respectively. A derivative (der) chromosome results from a translocation, and is numbered according to its centromere’s chromosome of origin (e.g. der19). Given a translocation between breakpoints on opposite arms, the genetic materials flip (black triangles) prior to re-attaching to their partner chromosomes.
Not drawn to scale


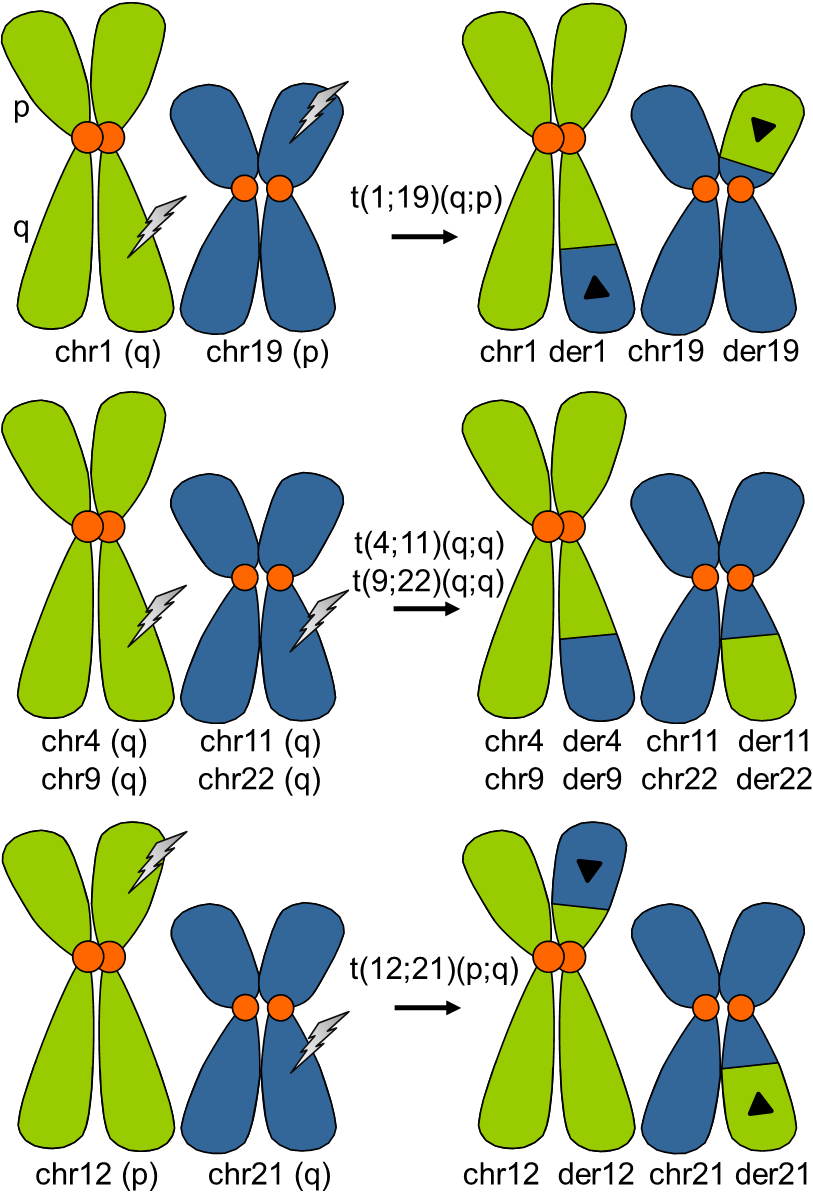


## Figure S2 - Simulation and experimental read length distributions

Read length distributions observed experimentally (left) and in simulation (right). We used a weighted mixture of log normal, Gaussian, and uniform read length distributions.


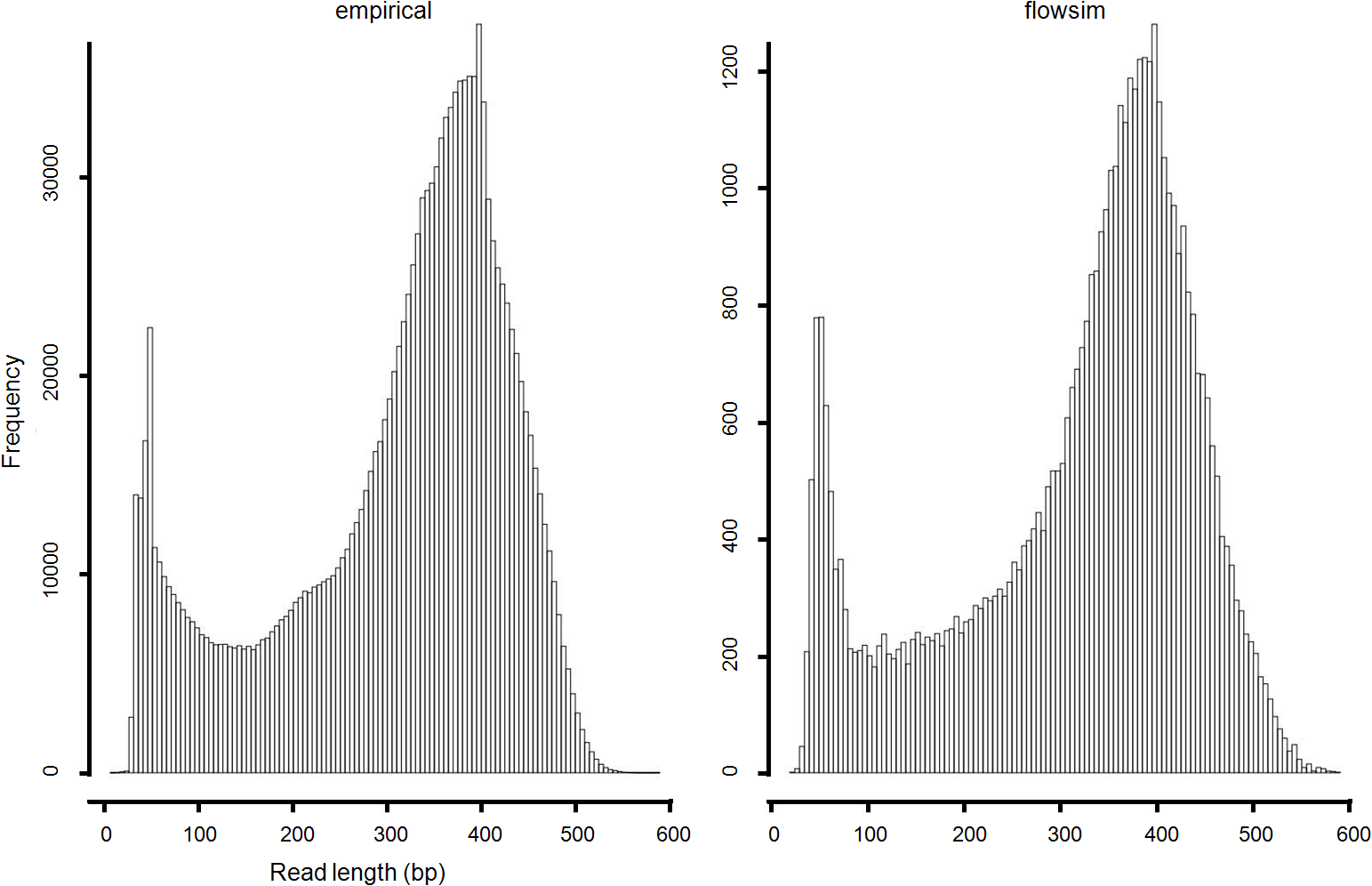


## Figure S3 - Capture probability of simulated DNA fragment given physical location and empirical coverages

Coverage was the average number of aligned reads spanning each physical location in the genomic regions in the capture target in samples 96C and 4. C_max_ was the maximum coverage value. We defined a simulated DNA fragment’s capture probability (p_capture_) as the sum of coverages of coordinates spanned by the DNA fragment divided by the product of C_max_ and the fragment length. Visually, the capture probability is the area of the dark grey region (AUC) divided by the total area of the rectangle defined by the DNA fragment and C_max_.
**AUC**: Area under curve, referring to sum of coverages spanned by the DNA fragment of interest;
**C_max_**: Maximum coverage value across all physical positions in capture target


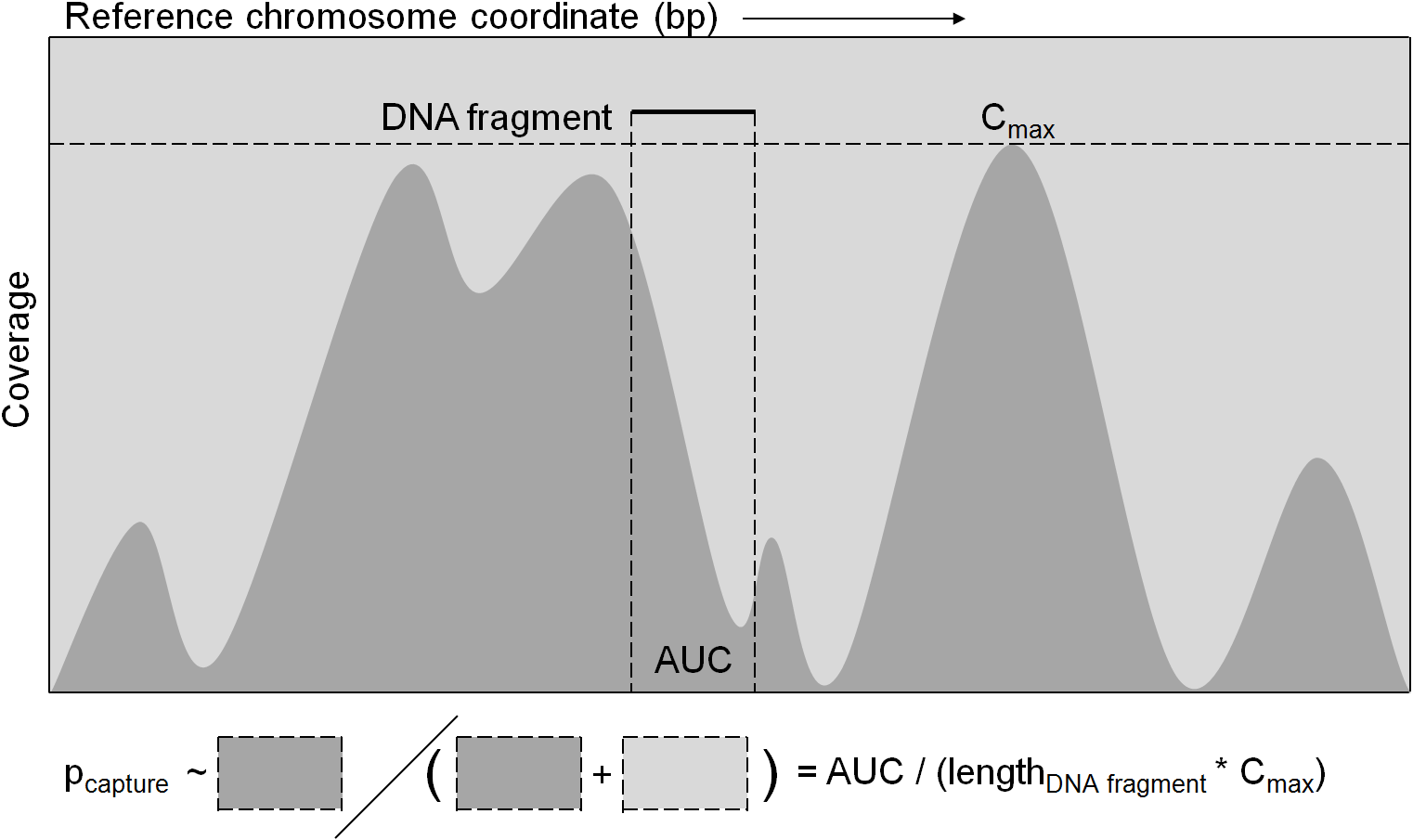


## Figure S4 - Capturing fragments of DNA spanning a junction by simulation

We treated SV-spanning fragments in order to approximate incomplete “hybridization.” The probability of capturing a junction-spanning fragment is approximated as the maximum probability from the two genomic regions if they were treated independently (See Figure S3).


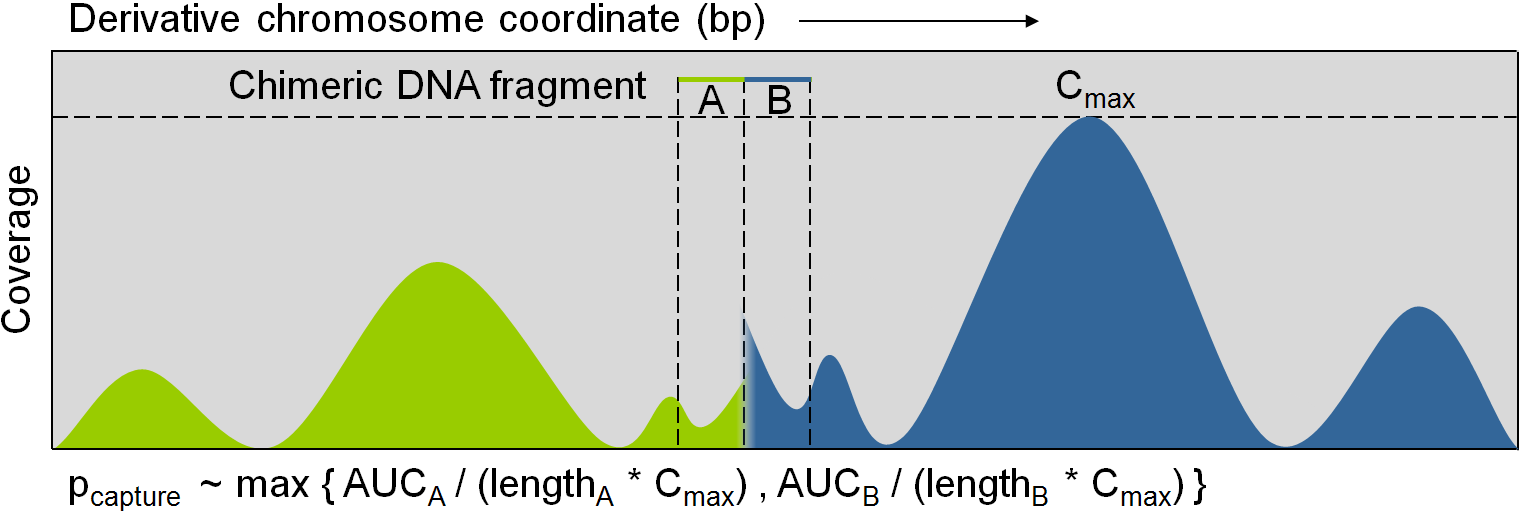


## Figure S5 - Simulated and experimental coverage values

Each of the 8 chromosomes in the capture target is shown separately. Each plotted point indicates the coverage for two samples at a physical location in the target. Each group of three plots shows (from top to bottom) the coverage relationship between experimental samples (96C and 4), then samples 4 and 96C with respect to a representative simulated sample on the x-axis.


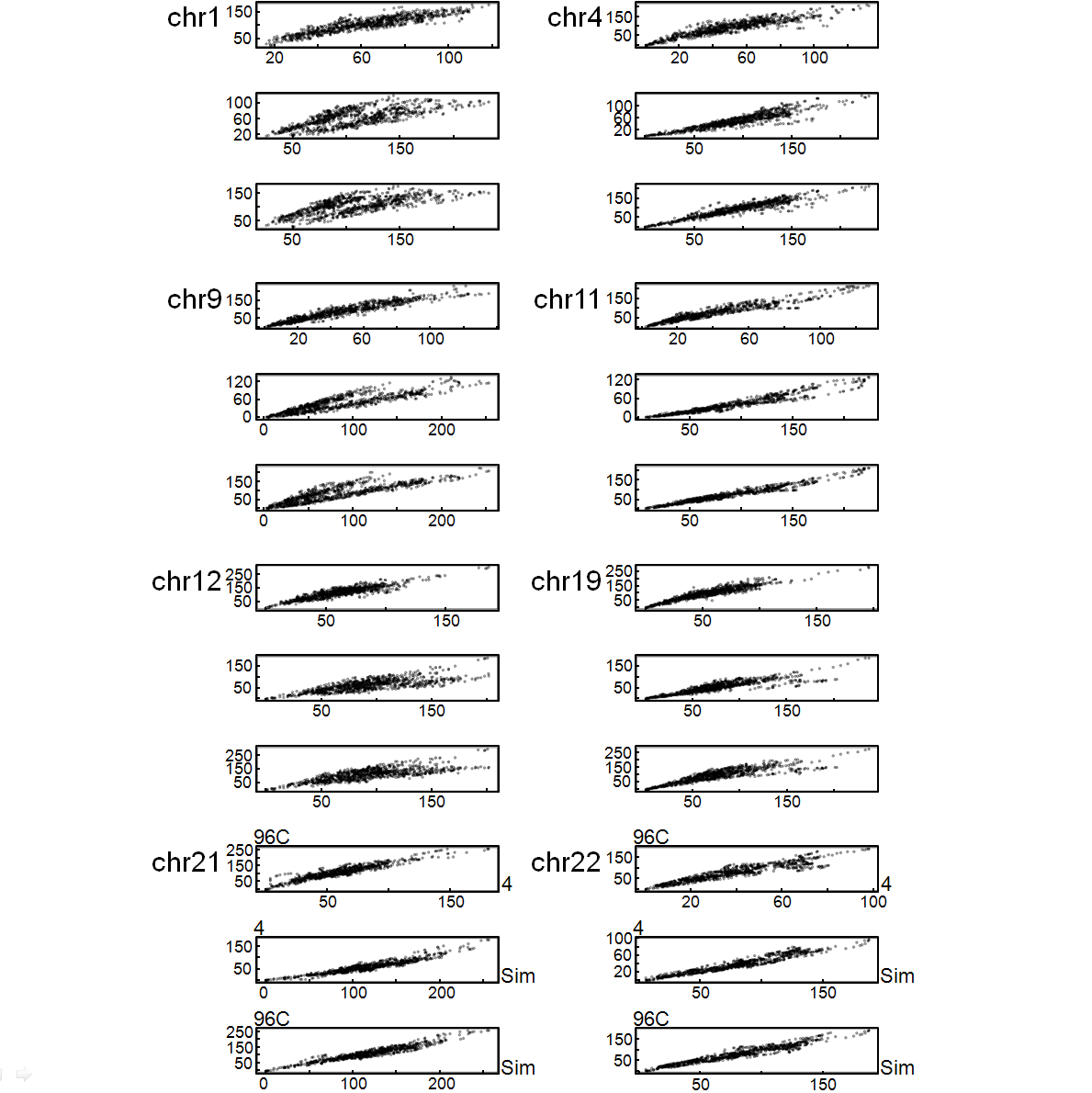


## Figure S6 - Observed and simulated base quality distributions

Confidence in accuracy of base calls [26-27] is shown as a function of position in read, as visualized by FastQC v0.9.6 [17] for sequencing data experimentally derived from enriched DNA fragments (left), and simulated by flowsim [14] (right). Base quality ($Q$) indicates the probability of an incorrect base call ($p$), where $p={10}^{-0.1Q}$. Base quality is shown in boxplot format, where a blue line represents mean quality, a red line indicates the median base quality, yellow boxes represent the interquartile range (25-75%), and the upper and lower whiskers represent the boundaries of the 90% and 10% percentiles, respectively. Medians of both empirical and simulated base quality distributions are largest until about position 150, beyond which there is gradual degradation.


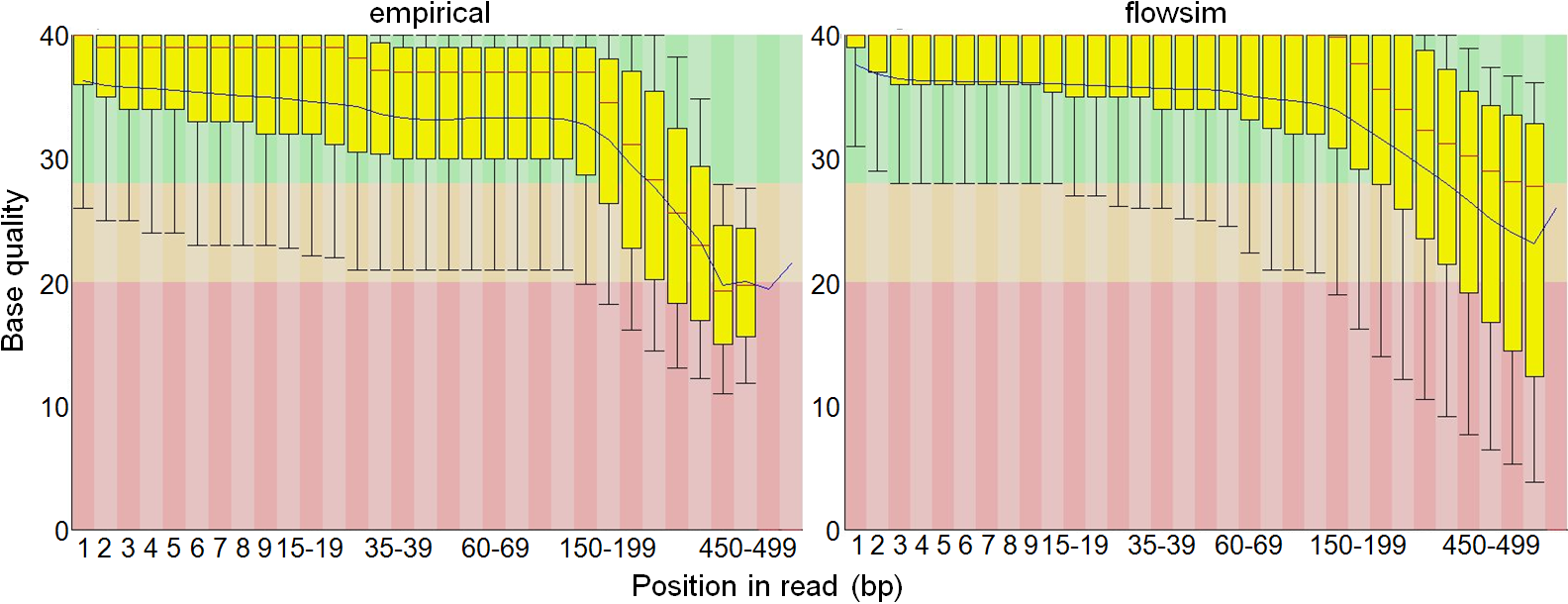


## Figure S7 - Detect interchromosomal rearrangements with SV-STAT

A translocation event (t(A;B)(q;q); box S7a) swaps materials between the q arms of chromosomes A (chrA; green) and B (chrB; blue), generating reciprocal SV (derivative chromosomes A and B; derA and derB; box S7b). The nucleotide sequences (reads) of chimeric fragments from derA and derB are aligned to the reference (box S7c). Reads sharing start or end coordinates “stack,” indicating candidate breakpoints in reverse or forward directions, respectively. Given a translocation between q arms of chrA and chrB, SV-STAT generates candidate junctions by concatenating reference sequences as illustrated next to derA and derB of (q;q) in the upper-right and lower-left quadrants of box S7d. The remainder of box S7d shows how SV-STAT generalizes for t(A;B)(q;p), (p;q), and (p;p). Next, SV-STAT measures support for the candidate junctions as illustrated in Figure 1.


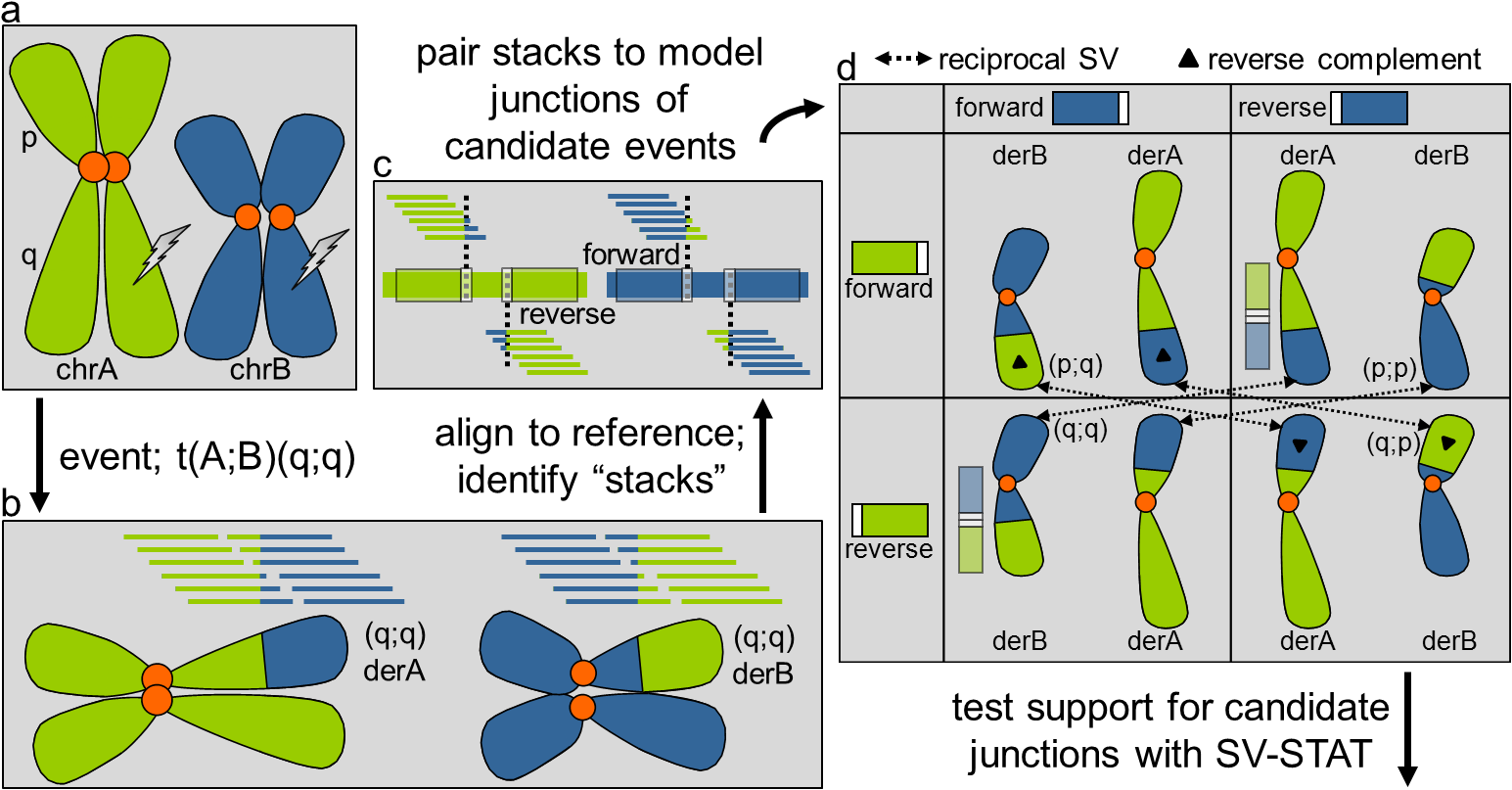


## Figure S8 - Specify type of intrachromosomal SV by order and orientation of DNA from paired breakpoint regions

DNA in the reference corresponds to ordered orange, green, blue, pink, and navy regions. Physical location (coordinate) increases from left to right. Pair forward and reverse stacks (defined in text and Figure S7) from breakpoint coordinates i (top and bottom, respectively) and j (left and right, respectively) as indicated to obtain a desired model. Arrows connect reciprocal junctions generated by the same underlying genomic rearrangement. Breakpoints are filled in white for junctions modeled by the combination of stacks indicated by the grid and transparent for the reciprocal SVs.
**i, j**: Physical locations of breakpoints of SVs, where i < j-1;
**1**: Inversion; **2**: Mobile element insertion; **3**: Interspersed duplication; **4**: Tandem duplication; **5**: Deletion


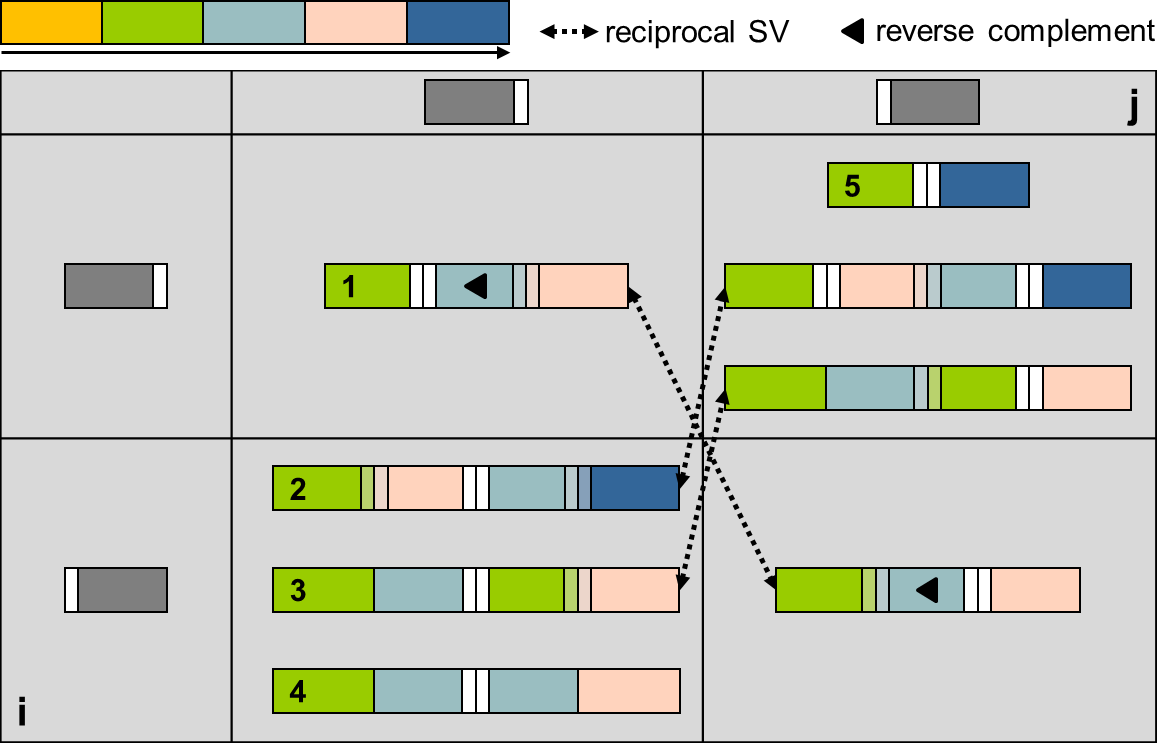


## Figure S9 - PCR validates SVs detected in samples 96C, 4, and 65C

After detecting SVs in sequence capture data, we ruled out the possibility that our findings were artifacts of library preparation. Polymerase chain reaction (PCR) amplified fragments of DNA spanning junctions for (**a**) an inversion in sample 96C (left), reciprocal translocations AFF1-MLL (der4) and MLL-AFF1 (der11) in sample 4 (right), and (**b**) reciprocal translocations PBX1-TCF3 (der1) and TCF3-PBX1 (der19) in sample 65C. We observed only nonspecific amplification using a sample of DNA from a healthy individual (N; NA17059) under otherwise identical conditions. Primer sequences are listed in Table S1.

**
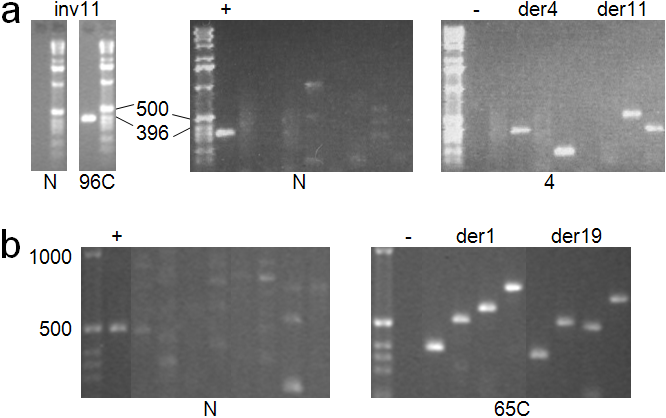
**

# Supplemental Tables

## Table S1 - Validation PCR primers

| **Primer name** | **Target** | **DNA sequence** |
| --- | --- | --- |
| 96C_1Mb_inv_SL | 96C_inv11 | CACCCCCAGGCATAGAAGAC |
| 96C_1Mb_inv_SR | 96C_inv11 | CAAGGCACCATTACACTTCC |
| CFD-I-04-96C-4.1F | chr4 | TAGGCACAGAGCATGCAAAC |
| CFD-I-04-96C-4.1R | chr4 | GCTACCTCTAGGATGAAAACTTGG |
| 4_der4_SL | 4_der4 | GCTTTTCACTTTCAGCAGACC |
| 4_der4_SR | 4_der4 | CAGAGGCCCAGCTGTAGTTC |
| 4_der4_LL | 4_der4 | TGGCTAATTTTTATATTGCTTTTGG |
| 4_der4_LR | 4_der4 | GACTACAGGTGCCCACCAC |
| 2796_2660_der11_LL | 4_der11 | TGGAAAGGACAAACCAGACC |
| 4_der11_SL | 4_der11 | CCAGTGGACTACTAAAACCCAAAG |
| 4_der11_SR | 4_der11 | AGATGAGTGGGGGAGAAATG |
| 4_der11_LR | 4_der11 | ACTCTCCTGGGCCTTTATGG |
| CFD-I-01-chr1F1 | chr1 | TATCCTTAAGCAGCCCATCG |
| CFD-I-01-chr1R1 | chr1 | TGGCAGGTTTTAGGTATTACAGG |
| CFD-I-13-65C-1.1F | 65C_der1 | ACGTGGGTCACAAAGAGGAG |
| CFD-I-13-65C-19.1F | 65C_der1 | AAACAGAGGGGAGCCTATGG |
| CFD-I-13-65C-19.2F | 65C_der1 | AGACCCCCGTACCCTGAG |
| CFD-I-13-65C-1.2F | 65C_der1 | GAACCACAGCCCATGCTATC |
| CFD-I-13-65C-1.1R | 65C_der19 | GTGTGACACCCTGTTCATGC |
| CFD-I-13-65C-19.1R | 65C_der19 | CCTGGGGATTGTTGAGTGTC |
| CFD-I-13-65C-19.2R | 65C_der19 | GCCCACAGGATTTGTGATG |
| CFD-I-13-65C-1.2R | 65C_der19 | GATTTCCCCTCCGTCCTC |

## Table S2 - Breakpoints of previously reported pre-B ALL translocations used for simulation

A list from TICdb [13] of translocation coordinates within the pre-B ALL breakpoint clusters of t(12;21) TEL-AML, t(1;19) E2A-PBX, t(9;22) BCR-ABL, and t(4;11) MLL-AF4 as determined in a number of primary studies [5-12]. Data from samples 32-33, 54-55, 59, and 67-68 were used to train SV-STAT’s detection threshold. Physical locations (coordinates) of breakpoints correspond to the March 2006 human genome assembly (hg18 [1]).

**Reference**: Unique nucleotide or publication identifier;
**JID**: Identifier for translocations used in this study;

**Sample**: Identifier for samples used in this study, some of which contained two translocations;
**SV**: Expected “derived” (der) chromosome numbered according to its centromere’s chromosome of origin;

**Cchr** and **Ccoord**: Physical location of breakpoint on the derived chromosome;

**Pchr** and **Pcoord**: Physical location of breakpoint on chromosome partnering with Cchr in the SV.

| **Reference** | **JID** | **Sample** | **SV** | **Cchr** | **Ccoord** | **Pchr** | **Pcoord** |
| --- | --- | --- | --- | --- | --- | --- | --- |
| 17889710 | 31 | 31 | der11 | chr11 | 117858421 | chr4 | 88231970 |
| AF029698 | 32 | 32-3 | der11 | chr11 | 117863455 | chr4 | 88215925 |
| AF029700 | 33 | 32-3 | der4 | chr4 | 88188827 | chr11 | 117860665 |
| AF031403 | 34 | 34 | der11 | chr11 | 117861555 | chr4 | 88203772 |
| AF177232 | 35 | 35-6 | der11 | chr11 | 117858885 | chr4 | 88228109 |
| AF177233 | 36 | 35-6 | der4 | chr4 | 88228356 | chr11 | 117858886 |
| AF177235 | 37 | 37-8 | der4 | chr4 | 88226942 | chr11 | 117858755 |
| AF487903 | 38 | 37-8 | der11 | chr11 | 117864340 | chr4 | 88194905 |
| AF487904 | 39 | 39-40 | der4 | chr4 | 88194884 | chr11 | 117864347 |
| AF492835 | 40 | 39-40 | der11 | chr11 | 117864526 | chr4 | 88222505 |
| AJ408891 | 41 | 41 | der11 | chr11 | 117858849 | chr4 | 88197200 |
| AJ408893 | 42 | 42 | der11 | chr11 | 117863003 | chr4 | 88188721 |
| AJ408894 | 43 | 43-4 | der11 | chr11 | 117863061 | chr4 | 88197666 |
| AJ408895 | 44 | 43-4 | der4 | chr4 | 88197612 | chr11 | 117863104 |
| 12415113 | 45 | 45 | der19 | chr19 | 1568930 | chr1 | 163026673 |
| 12415113 | 46 | 46 | der19 | chr19 | 1568930 | chr1 | 162938167 |
| 12415113 | 47 | 47 | der19 | chr19 | 1568927 | chr1 | 162985611 |
| 12415113 | 48 | 48 | der19 | chr19 | 1568927 | chr1 | 163010445 |
| 12415113 | 49 | 49 | der19 | chr19 | 1568927 | chr1 | 163022088 |
| 12415113 | 50 | 50 | der19 | chr19 | 1568932 | chr1 | 163020191 |
| 12415113 | 51 | 51 | der19 | chr19 | 1568928 | chr1 | 162940194 |
| 12415113 | 52 | 52-3 | der19 | chr19 | 1569177 | chr1 | 163026948 |
| 12415113 | 53 | 52-3 | der1 | chr1 | 163022376 | chr19 | 1568928 |
| 12415113 | 54 | 54-5 | der19 | chr19 | 1568931 | chr1 | 163022385 |
| 12415113 | 55 | 54-5 | der1 | chr1 | 163021922 | chr19 | 1568928 |
| U19398 | 56 | 56 | der22 | chr22 | 21963696 | chr9 | 132651934 |
| U19399 | 57 | 57 | der22 | chr22 | 21962844 | chr9 | 132717320 |
| U19400 | 58 | 58 | der22 | chr22 | 21963594 | chr9 | 132695453 |
| U19408 | 59 | 59 | der22 | chr22 | 21962443 | chr9 | 132604891 |
| 10992297 | 60 | 60-1 | der21 | chr21 | 35242284 | chr12 | 11920696 |
| 10992297 | 61 | 60-1 | der12 | chr12 | 11921108 | chr21 | 35204911 |
| 10992297 | 62 | 62 | der12 | chr12 | 11920966 | chr21 | 35239278 |
| 10992297 | 63 | 63-4 | der12 | chr12 | 11917437 | chr21 | 35181599 |
| 10992297 | 64 | 63-4 | der21 | chr21 | 35270131 | chr12 | 11920634 |
| 10992297 | 65 | 65-6 | der12 | chr12 | 11928175 | chr21 | 35186466 |
| 10992297 | 66 | 65-6 | der21 | chr21 | 35334005 | chr12 | 11924427 |
| 10992297 | 67 | 67-8 | der12 | chr12 | 11921176 | chr21 | 35238884 |
| 10992297 | 68 | 67-8 | der21 | chr21 | 35342615 | chr12 | 11921455 |

## Table S3 - Train SV-STAT detection threshold given Roche/454 sequencing data simulated from 4 samples with 7 previously observed translocations in pre-B ALL patients

The lowest-scoring true positive (**†**) and next-lowest prediction (**‡**) support scores were 3.02119 and 2.9489, respectively. Their average was 2.985045, which we defined as the threshold above which SV-STAT would predict SVs.

**Sample**: Identifier for samples used in this study, some of which contained two translocations;

**CandidateID**: Physical locations and orientations of breakpoints in the predicted structural variation. The two breakpoints are shown separated by an underscore, where each breakpoint’s chromosome, coordinate (hg18 [1]), and orientation values are separated by colons;

**log_10_(*S*)**: Support metric for candidate junction, as determined by SV-STAT;

**GS**: The gold standard indicating whether the junction was present in the simulation

| **Sample** | **CandidateID** | **log10(S)** | **GS** |
| --- | --- | --- | --- |
| 67-8 | chr21:35342615:+_chr12:11921455:- | 4.37561 | yes |
| 54-55 | chr1:163022387:-_chr19:1568930:+ | 4.1365 | yes |
| 67-8 | chr21:35238884:-_chr12:11921176:+ | 4.09767 | yes |
| 59 | chr22:21962443:+_chr9:132604887:+ | 3.75159 | yes |
| 32-33 | chr4:88188823:+_chr11:117860663:+ | 3.38021 | yes |
| 32-33 | chr11:117863455:+_chr4:88215924:+ | 3.35889 | yes |
| 67-8 | chr11:117882652:+_chr4:88167074:+ | 3.07041 | no |
| † 54-55 | chr1:163021923:+_chr19:1568925:- | 3.02119 | yes |
| ‡ 54-55 | chr22:21858725:+_chr9:132759338:+ | 2.9489 | no |
| 32-33 | chr4:88283840:+_chr11:117860663:+ | 2.91328 | no |
| 54-55 | chr21:35380123:+_chr12:11924799:- | 2.84136 | no |
| 67-8 | chr9:132567498:+_chr22:21800090:+ | 2.83378 | no |
| 54-55 | chr9:132572786:+_chr22:21789999:+ | 2.81757 | no |

## Table S4 - Predictions of structural variations (SVs) by SV-STAT given Roche/454 sequencing data simulated from 23 samples with 31 previously observed translocations in pre-B ALL patients

All candidate junctions with support scores above 2.985045 are predicted to be SVs by SV-STAT. Of the 46,874 candidates considered, only the 34 highest-scoring are shown. Rows representing false positive predictions are indicated with an asterisk in the first column.

**Sample**: Identifier for samples used in this study, some of which contained two translocations;
**SV**: Name of expected “derived” (der) chromosome following rearrangement;

**Achr**, **Acoord**, **Aori**, **Bchr**, **Bcoord**, **and Bori**: Physical locations (hg18) and orientations of the breakpoints connected by the SV, as predicted by SV-STAT. The letters A and B indicate the first and second breakpoints of the translocation, respectively, as viewed along the forward strand of the derivative chromosome;

**log_10_(*S*)**: Support metric for candidate junction, as determined by SV-STAT

| **Sample** | **SV** | **Achr** | **Acoord** | **Aori** | **Bchr** | **Bcoord** | **Bori** | **log10(S)** |
| --- | --- | --- | --- | --- | --- | --- | --- | --- |
| 50 | der19 | chr1 | 163020191 | - | chr19 | 1568932 | + | 4.76551 |
| 41 | der11 | chr11 | 117858849 | + | chr4 | 88197200 | + | 4.75183 |
| 47 | der19 | chr1 | 162985611 | - | chr19 | 1568927 | + | 4.64854 |
| 63-4 | der21 | chr21 | 35270131 | + | chr12 | 11920634 | - | 4.61946 |
| 62 | der12 | chr21 | 35239278 | - | chr12 | 11920966 | + | 4.58127 |
| 63-4 | der12 | chr21 | 35181599 | - | chr12 | 11917437 | + | 4.519 |
| 49 | der19 | chr1 | 163022088 | - | chr19 | 1568927 | + | 4.50376 |
| 37-8 | der4 | chr4 | 88226942 | + | chr11 | 117858755 | + | 4.4604 |
| 35-6 | der11 | chr11 | 117858885 | + | chr4 | 88228109 | + | 4.45096 |
| 56 | der22 | chr22 | 21963696 | + | chr9 | 132651934 | + | 4.40202 |
| 34 | der11 | chr11 | 117861555 | + | chr4 | 88203772 | + | 4.40012 |
| 60-1 | der12 | chr21 | 35204911 | - | chr12 | 11921108 | + | 4.37814 |
| 43-4 | der4 | chr4 | 88197610 | + | chr11 | 117863102 | + | 4.3662 |
| 35-6 | der4 | chr4 | 88228356 | + | chr11 | 117858886 | + | 4.27177 |
| 45 | der19 | chr1 | 163026673 | - | chr19 | 1568931 | + | 4.18879 |
| 39-40 | der4 | chr4 | 88194884 | + | chr11 | 117864345 | + | 4.1521 |
| 42 | der11 | chr11 | 117863003 | + | chr4 | 88188721 | + | 4.10261 |
| 39-40 | der11 | chr11 | 117864526 | + | chr4 | 88222505 | + | 4.05801 |
| 48 | der19 | chr1 | 163010444 | - | chr19 | 1568927 | + | 3.97923 |
| 43-4 | der11 | chr11 | 117863061 | + | chr4 | 88197664 | + | 3.84236 |
| 46 | der19 | chr1 | 162938167 | - | chr19 | 1568929 | + | 3.81803 |
| 60-1 | der21 | chr21 | 35242284 | + | chr12 | 11920697 | - | 3.73488 |
| 65-6 | der21 | chr21 | 35334006 | + | chr12 | 11924427 | - | 3.62242 |
| 57 | der22 | chr22 | 21962845 | + | chr9 | 132717320 | + | 3.58331 |
| 37-8 | der11 | chr11 | 117864340 | + | chr4 | 88194904 | + | 3.56074 |
| 51 | der19 | chr1 | 162940193 | - | chr19 | 1568928 | + | 3.43489 |
| 52-3 | der1 | chr1 | 163022376 | + | chr19 | 1568900 | - | 3.42651 |
| 58 | der22 | chr22 | 21963595 | + | chr9 | 132695453 | + | 3.31597 |
| * 49 | der22 | chr22 | 21873800 | + | chr9 | 132561223 | + | 3.16554 |
| 31 | der11 | chr11 | 117858421 | + | chr4 | 88231967 | + | 3.00043 |
| * 56 | der1 | chr1 | 162992998 | + | chr19 | 1585865 | - | 2.99564 |
| 34 | der22 | chr9 | 132648381 | + | chr22 | 21895329 | + | 2.96379 |
| 56 | der22 | chr9 | 132608013 | + | chr22 | 21869912 | + | 2.95521 |
| 57 | der1 | chr1 | 162952267 | + | chr19 | 1557276 | - | 2.95134 |

#

# Bibliography

1. Lander, E. S. *et al*. Initial sequencing and analysis of the human genome *Nature* **409**, 860-921 (2001).

2. Margulies, M. *et al*. Genome sequencing in microfabricated high-density picolitre reactors *Nature* **437**, 376-380 (2005).

3. Wheeler, D. A. *et al*. The complete genome of an individual by massively parallel DNA sequencing *Nature* **452**, 872-876 (2008).

4. Albert, T. J. *et al*. Direct selection of human genomic loci by microarray hybridization *Nat. Methods* **4**, 903-905 (2007).

5. Bizarro, S. *et al*. Molecular characterization of a rare MLL-AF4 (MLL-AFF1) fusion rearrangement in infant leukemia *Cancer Genet. Cytogenet.* **178**, 61-64 (2007).

6. Gale, K. B. *et al*. Backtracking leukemia to birth: identification of clonotypic gene fusion sequences in neonatal blood spots *Proc. Natl. Acad. Sci. U. S. A.* **94**, 13950-13954 (1997).

7. Felix, C. A. *et al*. Panhandle polymerase chain reaction amplifies MLL genomic translocation breakpoint involving unknown partner gene *Blood* **90**, 4679-4686 (1997).

8. Felix, C. A. *et al*. Duplicated regions of AF-4 intron 4 at t(4;11) translocation breakpoints *Mol. Diagn.* **4**, 269-283 (1999).

9. Raffini, L. J. *et al*. Panhandle and reverse-panhandle PCR enable cloning of der(11) and der(other) genomic breakpoint junctions of MLL translocations and identify complex translocation of MLL, AF-4, and CDK6 *Proc. Natl. Acad. Sci. U. S. A.* **99**, 4568-4573 (2002).

10. Reichel, M. *et al*. Biased distribution of chromosomal breakpoints involving the MLL gene in infants versus children and adults with t(4;11) ALL *Oncogene* **20**, 2900-2907 (2001).

11. Wiemels, J. L. *et al*. Site-specific translocation and evidence of postnatal origin of the t(1;19) E2A-PBX1 fusion in childhood acute lymphoblastic leukemia *Proc. Natl. Acad. Sci. U. S. A.* **99**, 15101-15106 (2002).

12. Zhang, J. G., Goldman, J. M. & Cross, N. C. Characterization of genomic BCR-ABL breakpoints in chronic myeloid leukaemia by PCR *Br. J. Haematol.* **90**, 138-146 (1995).

13. Novo, F. J., de Mendibil, I. O. & Vizmanos, J. L. TICdb: a collection of gene-mapped translocation breakpoints in cancer *BMC Genomics* **8**, 33 (2007).

14. Balzer, S., Malde, K., Lanzen, A., Sharma, A. & Jonassen, I. Characteristics of 454 pyrosequencing data--enabling realistic simulation with flowsim *Bioinformatics* **26**, i420-i425 (2010).

15. Cock, P. J., Fields, C. J., Goto, N., Heuer, M. L. & Rice, P. M. The Sanger FASTQ file format for sequences with quality scores, and the Solexa/Illumina FASTQ variants *Nucleic Acids Res.* **38**, 1767-1771 (2010).

16. [http://seqanswers.com/forums/showpost.php?p=29075&postcount=8](http://seqanswers.com/forums/showpost.php?p=29075&postcount=8).

17. [http://www.bioinformatics.babraham.ac.uk/projects/fastqc/](http://www.bioinformatics.babraham.ac.uk/projects/fastqc/).

18. Li, H. & Durbin, R. Fast and accurate long-read alignment with Burrows-Wheeler transform *Bioinformatics* **26**, 589-595 (2010).

19. Li, H. *et al*. The Sequence Alignment/Map format and SAMtools *Bioinformatics* **25**, 2078-2079 (2009).

20. [http://compbio.dfci.harvard.edu/tgi/software/](http://compbio.dfci.harvard.edu/tgi/software/).

21. Quinlan, A. R. & Hall, I. M. BEDTools: a flexible suite of utilities for comparing genomic features *Bioinformatics* **26**, 841-842 (2010).

22. Stajich, J. E. *et al*. The Bioperl toolkit: Perl modules for the life sciences *Genome Res.* **12**, 1611-1618 (2002).

23. [http://picard.sourceforge.net/](http://picard.sourceforge.net/).

24. Alkan, C., Coe, B. P. & Eichler, E. E. Genome structural variation discovery and genotyping *Nature Reviews Genetics* (2011).

25. Pages, H. BSgenome: Infrastructure for Biostrings-based genome data packages. R package version 1.24.0

26. Ewing, B., Hillier, L., Wendl, M. C. & Green, P. Base-calling of automated sequencer traces using phred. I. Accuracy assessment *Genome Res.* **8**, 175-185 (1998).

27. Ewing, B. & Green, P. Base-calling of automated sequencer traces using phred. II. Error probabilities *Genome Res*. **8**, 186-194 (1998).

28. Wang, J. et al. *Nat. Methods* **8**, 652-654 (2011).

29. Chen, K. et al. BreakDancer: an algorithm for high-resolution mapping of genomic structural variation. *Nat. Methods* **6**, 677-681 (2009).
